# HPV self-sampling for cervical cancer screening: a systematic review of values and preferences

**DOI:** 10.1136/bmjgh-2020-003743

**Published:** 2021-05-19

**Authors:** Holly Nishimura, Ping Teresa Yeh, Habibat Oguntade, Caitlin E Kennedy, Manjulaa Narasimhan

**Affiliations:** 1 Department of International Health, Johns Hopkins University Bloomberg School of Public Health, Baltimore, Maryland, USA; 2 Department of Sexual and Reproductive Health and Research, UNDP-UNFPA-UNICEF-WHO-World Bank Special Programme of Research, Development and Research Training in Human Reproduction (HRP), Geneva, Switzerland

**Keywords:** systematic review, public health, cancer, other infection, disease, disorder, or injury

## Abstract

**Introduction:**

The WHO recommends human papillomavirus (HPV) cervical self-sampling as an additional screening method and HPV DNA testing as an effective approach for the early detection of cervical cancer for women aged ≥30 years. This systematic review assesses end user’s values and preferences related to HPV self-sampling.

**Methods:**

We searched four electronic databases (PubMed, Cumulative Index to Nursing and Allied Health Literature, Latin American and Caribbean Health Sciences Literature and Embase) using search terms for HPV and self-sampling to identify articles meeting inclusion criteria. A standardised data extraction form was used to capture study setting, population, sample size and results related to values and preferences.

**Results:**

Of 1858 records retrieved, 72 studies among 52 114 participants published between 2002 and 2018 were included in this review. Almost all studies were cross-sectional surveys. Study populations included end users who were mainly adolescent girls and adult women. Ages ranged from 14 to 80 years. Most studies (57%) were conducted in high-income countries. Women generally found HPV self-sampling highly acceptable regardless of age, income or country of residence. Lack of self-confidence with collecting a reliable sample was the most commonly cited reason for preferring clinician-collected samples. Most women preferred home-based self-sampling to self-sampling at a clinic. The cervical swab was the most common and most accepted HPV DNA sampling device.

**Conclusions:**

HPV self-sampling is generally a highly accepted method of cervical cancer screening for end users globally. End user preferences for self-sampling device, method and setting can inform the development of new and expanded interventions to increase HPV screening.

**Key questions**

**What is already known?**

- Self-sampling for human papillomavirus (HPV) DNA is effective at detecting cervical cancer and generally a highly acceptable form of cervical cancer screening to end users.

**What are the new findings?**

- Self-sampled specimens are seen as acceptable in terms of ease of use, convenience, privacy, and physical and emotional comfort (including decreased embarrassment, anxiety, and pain).
- Preferences related to self-sampling including sampling device (eg, swab, brush and tampon), method (eg, cervicovaginal and urine), setting (eg, home, clinic and community-based site) varied by region, subpopulation and age, though most end users found the process of self-sampling acceptable overall.

**What do the new findings imply?**

- End user preferences for self-sampling method, device and setting can inform the development of new and expanded interventions to increase HPV screening.
- Understanding end users’ preferences for self-sampling is critical for reaching the WHO global target of 70% HPV screening coverage by 2030.

## Introduction

Cervical cancer is the fourth most common cancer among women globally, with the estimated 570 000 new cases yearly mostly affecting women between the ages of 30 and 49 years.^1^ This preventable cancer causes a woman to die every 2 min, with 90% of 311 000 annual deaths occurring in low-income and middle-income countries.^2^ Cervical cancer is caused by certain types of human papillomavirus (HPV), with HPV-16 and HPV-18 subtypes causing 70% of cervical cancers and precancerous cervical lesions.^3^ Evidence-based and cost-effective cervical cancer prevention includes primary prevention with HPV vaccination. Many successful national HPV vaccination programmes have been introduced in high-income and upper middle-income countries, but HPV vaccine introduction in low-income and middle-income countries (LMICs) remains insufficient.^4^ Therefore, secondary prevention with early, low-cost, high-quality HPV DNA screening is essential to reduce mortality and morbidity from cervical cancer in LMICs. Increasing access to and acceptability of cervical cancer screening is in line with the WHO’s strategy for cervical cancer elimination, which includes targets to achieve a global coverage of 90% vaccination, 70% screening and 90% treatment by 2030.^5 6^

HPV DNA testing is recommended by WHO, the US Preventive Services Task Force and Australian, US and European national screening programme guidelines as an effective approach for the early detection of cervical cancer for women ≥30 years.^7–9^ In addition, in areas with high endemic HIV, WHO recommends that sexually active girls and women, regardless of age, should be screened as soon as they have tested positive for HIV.^7^ Typically, per current recommendations, cervicovaginal samples for HPV DNA testing are collected by a clinician during a pelvic examination.^8 9^ However, cervicovaginal HPV DNA specimens can also be reliably collected by end users themselves—a process known as self-sampling.^10–12^ Generally, HPV DNA self-sampling is a highly acceptable method for the purposes of cervical cancer screening^13^ and has been associated with improved participation in cervical cancer screening studies in low-income and middle-income countries.^14^

Although previous reviews have found generally high acceptability of HPV DNA self-sampling, end users’ values and preferences regarding self-sampling method, device and setting have not been explored.^13 15–17^ The purpose of this systematic review is to synthesise the literature on end users’ values and preferences related to HPV DNA self-sampling for the purposes of cervical cancer screening. Values and preferences are defined according to Guyatt *et al* as the ‘collection of goals, expectations, predispositions, and beliefs that individuals have for certain decisions and their potential outcomes’.^18^ The values and preferences from this systematic review were used as part of the evidence base for the 2019 WHO Consolidated Guideline on Self-Care Interventions for Health.^19^ Understanding end users’ values and preferences related to self-sampling is critical because HPV testing must be acceptable to end users, in particular those who are underscreened and most vulnerable to cervical cancer. This review is further intended to support the WHO latest guidance on HPV screening, including to programme managers within the national cervical cancer prevention and control programmes.^20^

## Methods

We conducted this systematic review in accordance with the Preferred Reporting Items for Systematic Reviews and Meta-Analyses guidelines.^21^

### Inclusion criteria

We defined HPV self-sampling as a process where a client who wants to know whether they have an HPV infection uses a kit to collect a cervicovaginal or urine sample, which is then sent for analysis by a laboratory. While self-sampled anal specimens can be collected for the detection of HPV DNA, these samples are likely to be indicative of risk for anal cancer rather than cervical cancer. Therefore, we only included articles that focused on cervicovaginal and urine samples given our interest in cervical cancer. Collection devices include lavage, brush, swab, tampon or labial padette and may occur in any setting (eg, home, community and clinic). We defined HPV clinician sampling as any sampling method where a clinician or other healthcare provider obtains the cervicovaginal HPV DNA sample. HPV DNA testing does not provide a positive diagnosis for cervical cancer but rather identifies those end users at risk for developing cervical cancer in the future.

Studies were eligible for inclusion if they met the following criteria: (1) included participants who performed cervicovaginal or urine self-sampling for HPV DNA; (2) measured general acceptability or characteristics of acceptability regarding cervicovaginal or urine self-sampling for cervical cancer or preference for setting for sampling (eg, home, community or clinic setting); and (3) published in a peer-reviewed journal prior to the search date. Both qualitative and quantitative studies were included.

### Search strategy and screening process

We searched four electronic databases (PubMed, the Cumulative Index to Nursing and Allied Health Literature, Latin American and Caribbean Health Sciences Literature and Embase) through 19 October 2018 using search terms for HPV and self-sampling. The full search strategy is described in a complementary review of the effectiveness of HPV self-sampling compared with clinician sampling^22^; the same search strategy was used for both the effectiveness review and this values and preferences review.

Articles were screened based on relevance to each topic. Ongoing randomised controlled trials (RCTs) were hand-searched for through ClinicalTrials.gov, the WHO International Clinical Trials Registry Platform, the Pan African Clinical Trials Registry and the Australian New Zealand Clinical Trials Registry. We conducted secondary searching of included articles and relevant reviews for additional studies that met the inclusion criteria. After initial title–abstract screening, full-text articles were obtained of all potential studies. Two reviewers independently assessed all full-text articles for study inclusion eligibility and resolved differences through consensus.

### Data extraction and analysis

Two reviewers independently used a standardised data abstraction form to capture information on location of study, study population, sample size and results related to values and preferences from each study. Differences in data abstraction were resolved through consensus, with a third reviewer as needed.

For studies that presented quantitative data related to values and preferences, two reviewers extracted measures of acceptability and satisfaction and their ratings from the results related to values and preferences.

For qualitative studies, we used an iterative approach to identify salient themes related to end users’ values and preferences. Codes were created based on recurring themes and applied to each article. One member of the study team read through each article and extracted themes inductively related to values and preferences for self-sampling. The study team met throughout the analysis process to discuss themes.

We also stratified findings by end users’ age, location of study, self-sampling device, setting and subpopulation (eg, women living with HIV, sexual and gender minorities) to examine differences in values and preferences by these characteristics.

A coding matrix was modified from the Evidence Project risk of bias tool^23^ and the Critical Appraisal Skills Program checklist for qualitative studies to facilitate data extraction related to quality assessment. The matrix included fields related measurement bias (eg, representativeness and missingness), sampling or selection bias and generalisability.

### Patient and public involvement

Members of the WHO patient safety working group provided feedback on the review during conceptualisation and protocol development. Patients were involved in a global survey of values and preferences conducted to inform the WHO guideline on self-care interventions; they thus play a significant role in the overall recommendation informed by this review.

## Results

**Figure 1:**
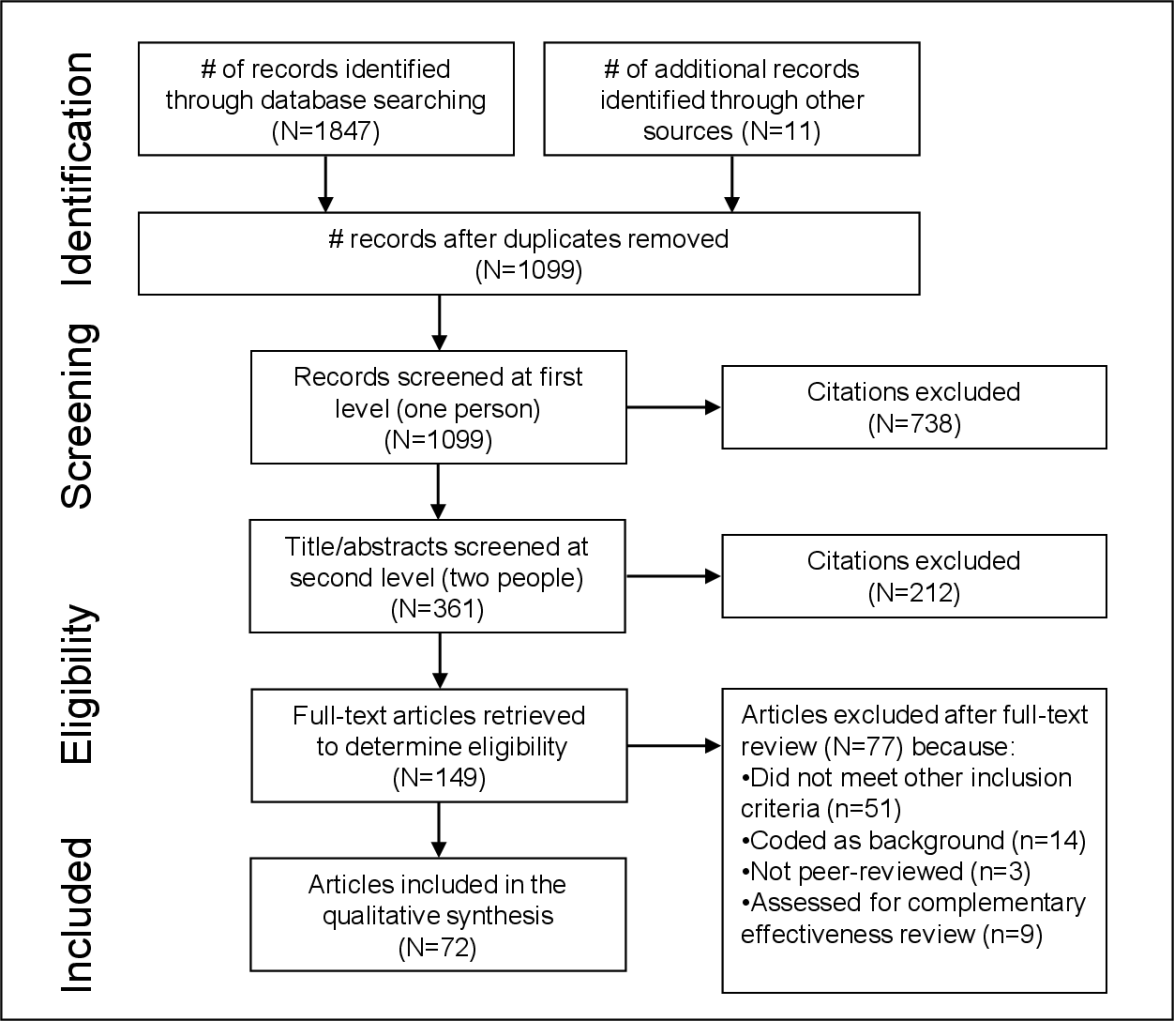
PRISMA flow diagram of the different phases of a systematic review. PRISMA, Preferred Reporting Items for Systematic Reviews and Meta-Analyses.

We retrieved 1847 records via electronic databases, with 11 additional citations reviewed from references listed in prior reviews, included studies, and hand-searches. After removing duplicate references and screening citations, we identified 72 unique studies presenting values and preferences data on HPV self-sampling (figure 1).

### Characteristics of included studies

Table 1 presents summary characteristics of the 72 included studies, with more details per included study in table 2. The 72 studies included a total of 52 114 participants; sample sizes for individual studies ranged from 17 to 9484. Articles were published between 2002 and 2018, with 50% published since 2015. Studies were conducted in a variety of countries, including all WHO regions and all World Bank country income classification categories (high, middle and low income). Of the included participants, 57% were from high-income countries. Almost all studies were cross-sectional surveys, though some study designs included in-depth interviews and/or focus group discussions.^24–32^ Several values and preferences studies were nested within larger studies (often RCTs).^31–40^

**Table 1:** Summary description of included studies

Characteristic	Articles*
**Region**	
Africa: Cameroon, Gambia, Ghana, Kenya, Malawi, Nigeria, South Africa and Uganda	12
Asia: China, Japan, Laos, Malaysia and Thailand	12
Europe: France, Germany, Italy, The Netherlands, Portugal, Sweden, Switzerland and UK	13
Americas: Argentina, Brazil, Canada, Chile, El Salvador, Guatemala, Mexico, Nicaragua, Peru, Puerto Rico and USA	32
Australia/Pacific	2
Multiple regions (India, Nicaragua and Uganda)	1
**Populations (not mutually exclusive**)	
Women from the general population	63
Never-screened or underscreened	14
Women living with HIV	3
Female sex workers	1
Women experiencing homelessnes	1
Adolescent girls and young women	3
Sexual and gender minorities	2
**Study design**	
Qualitative	9
Quantitative	58
Mixed methods or multimethod	5
**Specimen collection device and methods**	
Swab	23
Brush	20
Lavage	3
Labial padette	1
Tampon†	5
Multiple devices	7
Urine‡	2
Unspecified	11
**Setting for self-sampling**	
Actual use at clinic	33
Actual use in workplace	1
Actual use at home	21
Actual use in community setting	4
Unspecified	13
**Total**	**72**

*The number of studies within each category is not mutually exclusive.

†All five studies compared tampon with other self-sampling devices.^32 34 49–51^

‡One study included both urine self-sampling and self-sampling using a cervical swab.^66^

**Table 2:** Characteristics of included studies

First author, year	Self-sampling device(s)	Hypothetical or actual use	Setting	Location	Population	Sample size	Study design
Abuelo,^71^ 2014	Unspecified	Actual	Community	Peru: Iquitos	Women aged 30–45 years and daughters or other girls aged 10–13 years in the community.	320	Quantitative
Aiko,^72^ 2017	Brush	Actual	Home	Japan: Yokohama	Women aged 20–69 with abnormal cervical cytology.	136	Quantitative
Andersson,^66^ 2018	Swab; urine	Actual	Clinic	Sweden: Stockholm	Women who had undergone treatment of high-grade CIN2+.	479	Quantitative
Anhang,^57^ 2005	Swab	Actual	Clinic	USA: New York, New York	Women aged ≥25 years who have not had a Pap test in the last year from four publicly funded clinics.	172	Quantitative
Arrossi,^69^ 2016	Brush	Actual	Home	Argentina: Jujuy	Women aged ≥30 years.	2616	MM
Bansil,^70^ 2014	Brush	Actual	Clinic	India, Nicaragua and Uganda	Women enrolled in the Screening Technologies to Advance Rapid Testing Utility and Program Planning project.	3863	MM
Berner,^73^ 2013	Swab	Actual	Clinic	Cameroon	Women aged 24–69 years.	243	Quantitative
Bosgraaf,^33^ 2014	Brush; lavage	Actual	Home	Netherlands	Women aged 29–63 years who are part of PROHTECT 3B trial.	9484	Quantitative
Cadman, 67 2015	Brush; swab	Hypothetical	n/a	UK: London	Hindu women aged 25–64 years.	185	MM
Castell,^74^ 2014	Lavage	Actual	Home	Germany: Hamburg, Hanover	Women aged 20–69 years within the German National Cohort.	108	Quantitative
Chen,^75^ 2014	Unspecified	Hypothetical	n/a	Taiwan	Women aged 18–65 years.	500	Quantitative
Crofts,^65^ 2015	Swab	Actual	Clinic	Cameroon: Tiko, Yaounde	Women aged 30–65 years.	540	Quantitative
Dareng,^76^ 2015	Unspecified	Hypothetical	n/a	Nigeria: Ondo, Abuja	Women aged ≥18 years	600	Quantitative
Dzuba,^77^ 2002	Swab	Actual	Clinic	Mexico	Women aged ≥20 years who use the Mexican Institute of Social Services, and who were involved in the parent study.	1069	Quantitative
Esber,^41^ 2017	Swab	Hypothetical	n/a	Malawi: Lilongwe	Women aged 15–39 years.	824	Quantitative
Fargnoli 24 2015	Swab	Hypothetical	n/a	Switzerland: Geneva	Women aged 24–67 years who had not been screened for cervical cancer in the last 3 years.	125	Qualitative
Flores,^78^ 2003	Swab	Actual	Clinic	Mexico: Morelos	Women aged 20–80 years using cervical cancer screening services at 23 health units that make up the Mexican National Cervical Cancer Screening Program.	7872	Quantitative
Gottschlich,^58^ 2017	Swab	Actual	Home	Guatemala: Santiago Atitlan	Indigenous women aged 18–60 years.	202	Quantitative
Guan,^53^ 2012	Brush	Actual	Clinic	China: Xiangyuan County	Women aged 30–58 years from the national cervical cancer screening programme.	174	Quantitative
Hanley,^79^ 2016	Brush	Actual	Workplace	Japan: Sapporo	Working women aged 20–49 years attending their annual workplace check-up at Sapporo Industrial Health Management Screening Center.	203	Quantitative
Harper,^34^ 2002a	Swab; tampon	Actual	Home	USA: New Hampshire	Women age 18–68 years attending Dartmouth teaching clinics who had been referred for a colposcopy for either abnormal Pap smear findings or abnormal cervical cancer examination findings.	103	Quantitative
Harper,^49^ 2002b	Swab; tampon	Actual	Clinic	USA: New Hampshire	Women aged 18–68 years who attended an affiliated Dartmouth-Hitchcock teaching clinic with abnormal Pap smear findings or abnormal cervical cancer findings.	103	Quantitative
Howard,^25^ 2009	Unspecified	Hypothetical	n/a	Canada: Hamilton, Ontario:	Married or previously married women aged 35–65 years who were a part of one of the following: English-speaking Canadian with low SES, and Arabic, Cantonese, Dari, Somali or Latina women who recently immigrated to Canada.	87	Qualitative
Igidbashian,^80^ 2011	Brush; lavage	Actual	Clinic	Italy: Milan	Women aged 19–72 years undergoing an excisional procedure for CIN.	205	Quantitative
Ilangovan,^42^ 2016	Brush	Actual	Clinic	USA: Miami, Florida	Haitian and Latina women aged 30–65 years who reported not receiving a Pap test in the last 3 years.	180	Quantitative
Jones,^81^ 2008	Lavage	Actual	Home	The Netherlands	Women aged 30–60 years attending 3 obstetrics and gynaecology hospital departments in Veldhoven, Arnhem and Tilburg, in the Netherlands.	104	Quantitative
Kahn,^64^ 2005	Swab	Actual	Clinic	USA: Ohio	Sexually active adolescent girls and young women aged 14–21 years attending an urban, hospital-based teen health centre.	121	Quantitative
Katz,^26^ 2017	Brush; lavage; swab	Hypothetical	Home	USA: Appalachian Ohio	Rural women aged 30–65 years who had not received a Pap test in the past 3 years.	15	Qualitative
Ketelaars,^82^ 2017	Brush	Actual	Home	The Netherlands	Women aged 30–60 years old participating in the Dutch screening programme.	2460	Quantitative
Kilfoyle,^43^ 2018	Brush	Actual	Home	USA: North Carolina	Low-income women overdue for cervical cancer screening.	227	Quantitative
Lack,^50^ 2005	Swab; tampon	Actual	Community	Gambia	Rural women.	377	Quantitative
Leniz,^44^ 2013	Brush	Actual	Clinic	Chile: Santiago	Women aged 30–64 years who reported not receiving a Pap test in the last 3 years.	1085	Quantitative
Levinson,^83^ 2013	Unspecified	Actual	Community	Peru: Manchay, Iquitos	Women aged 30–45.	643	Quantitative
Lindell,^45^ 2012	Swab	Actual	Home	Sweden	Women aged 50–65 years who had not attended cervical cancer screening in the last 6 years.	3618	Quantitative
Mahomed,^51^ 2014	Brush; lavage; tampon	Hypothetical	n/a	South Africa	HIV-positive women aged >18 years from urban and rural HIV clinics.	106	Quantitative
Mao,^35^ 2017	Swab	Actual	Home	USA: Seattle, Washington	Women aged 21–65 years attending a University of Washington clinic.	1769	Quantitative
Ma’som,^95^ 2016	Brush	Actual	Clinic	Malaysia: Selangor	Women aged 18–60 years attending 5 government-run general practice clinics.	839	Quantitative
Maza,^46^ 2018	Unspecified	Actual	Home	El Salvador: Paracentral region	Women aged 30–69 years who reported not receiving a Pap test in the last 3 years or HPV test in the last 5 years and had not undergone procedures associated with cervical intraepithelial neoplasia.	1867	Quantitative
Mbatha,^54^ 2017	Brush; swab	Actual	Clinic	South Africa: KwaZulu-Natal	Sexually active adolescent girls and young women aged 16–22 years enrolled in government high school.	91	Quantitative
McLachlan,^47^ 2018	Unspecified	Actual	Clinic	Australia: Victoria	Women aged 27–74 years who were underscreened or never screened.	40	MM
Mitchell,^61^ 2017	Swab	Actual	Clinic	Uganda	HIV-positive women aged 30–69 years.	87	Quantitative
Modibbo,^36^ 2017	Swab	Actual	Home	Nigeria: Abuja	Women aged 30–65 years.	400	Quantitative
Molokwu,^37^ 2018	Swab	Actual	Home	USA: El Paso County, Texas	Women aged 30–65 years with no cervical screening in the last 3 years.	202	Quantitative
Moran,^84^ 2017	Brush	Actual	Home	Peru: Ventanilla Callao	Women enrolled in the HOPE programme.	97	Quantitative
Nobbenhuis,^85^ 2002	Lavage	Actual	Home	Netherlands: Rotterdam and Amsterdam	Women aged 20–63 years.	56	Quantitative
Obiri-Yeboah,^86^ 2017	Brush	Actual	Clinic	Ghana: Cape Coast	Every fifth woman aged ≥18 years attending a teaching hospital.	194	Quantitative
Oranratanaphan 94 2014	Brush	Actual	Clinic	Thailand: Bangkok	Women aged 30–65 years.	100	Quantitative
Ortiz,^87^ 2012	Brush	Actual	Clinic	Puerto Rico	Women aged 18–34 years attending an OBGYN clinic for routine Pap test.	100	Quantitative
Penaranda,^27^ 2014	Unspecified	Hypothetical	n/a	US–Mexico border	Women aged 30–65 years.	21	Qualitative
Phoolcharoen,^88^ 2018	Brush	Actual	Clinic	Thailand: Bangkok	Women aged 30–70 years.	250	Quantitative
Pieters,^29^ 2013	Labial padette	Actual	Clinic	USA: Los Angeles, California	Homeless women aged 32–70 years.	17	Qualitative
Podolak,^68^ 2017	Unspecified	Hypothetical	n/a	Kenya: Nairobi	Women in counties near the University of Nairobi.	107	MM
Quincy,^89^ 2012	Brush; swab	Actual	Clinic	Nicaragua: Leon	Women aged 25–60 years.	250	Quantitative
Racey,^38^ 2016	Swab	Actual	Home	Canada: Ontario	Underscreened rural women aged 30–70 years.	100	Quantitative
Reisner,^62^ 2018	Swab	Actual	Clinic	USA: Boston, Massachusetts	Sexually active transgender males aged 21–64 years.	150	Quantitative
Reiter,^63^ 2015	Unspecified	Hypothetical	n/a	USA	Lesbian and bisexual women aged 21–26 years.	418	Quantitative
Rodrigues,^60^ 2018	Brush	Actual	Clinic	Brazil: Tapájos region	Women aged 17–75 years recruited from health centres; some women HIV+.	153	Quantitative
Rosenbaum,^52^ 2014	Brush	Actual	Clinic	El Salvador: Paracentral region	Women aged 30–49 years.	518	Quantitative
Silva,^90^ 2017	Unspecified	Actual	Unspecified	Portugal	Women aged 18–66 years.	313	Quantitative
Sy,^30^ 2017	Urine	Actual	Clinic	Federated States of Micronesia: Yap State	Women aged 21–65 years.	217	Qualitative
Szarewski,^28^ 2009	Lavage; swab	Hypothetical	n/a	UK: London	Muslim women aged 21–65 years recruited from the Noor Ul Islam Trust.	28	Qualitative
Tisci,^55^ 2003	Brush	Actual	Clinic	China	Women aged 35–60 years who may not have been screened for cervical neoplasia in the past 10 years.	1560	Quantitative
Trope,^56^ 2013	Swab	Actual	Community	Thailand: Roi-it Province	Women aged 25–60 years who had not been screened for cervical cancer in the past 5 years.	431	Quantitative
van de Wijgert,^32^ 2006	Swab; tampon	Actual	Clinic	South Africa: Cape Town	Sexually active women >18 years.	450	Qualitative
Vanderpool,^48^ 2014	Brush	Actual	Clinic	USA: Appalachian Kentucky	Women aged 30–64 years with no cervical screening in the last 3 years.	31	Quantitative
Waller,^91^ 2006	Swab	Actual	Clinic	UK: Central and West London	Women.	902	Quantitative
Winer,^59^ 2016	Swab	Actual	Home	USA: Hopi Reservation, Arizona	Hopi women aged 21–65 years.	353	Quantitative
Wong,^39^ 2016	Swab	Actual	Clinic	Hong Kong	Women aged 35–65 years.	392	Quantitative
Wong,^40^ 2018	Swab	Actual	Clinic	Hong Kong	Female sex workers aged ≥18 years.	68	Quantitative
Yoshida,^92^ 2013	Brush	Actual	Home	Laos	Women aged 18–80 years who worked in Khammouane Provincial Hospital, Xebangfay District Health Office or lived in Tung village in Sibounhouane subdistrict.	290	Quantitative
Zehbe,^93^ 2011	Swab	Actual	Home	Canada: Ontario	Women aged 25–59 years.	49	Quantitative
Zehbe,^31^ 2017	Swab	Hypothetical	n/a	Canada: Ontario	First Nation women aged ≥18 without formal postsecondary education.	85	Qualitative

CIN, Cervical Intraepithelial Neoplasia; HPV, human papillomavirus; SES, socioeconomic status.

Study populations typically included adult end users. Participants ranged in age from 14 to 80 years. Fourteen studies specifically targeted women who were under/never screened for cervical cancer.^24 26 30 31 37 38 41–48^ Others selected participants from specific subgroups or vulnerable populations, including women from rural areas,^26 38 41 48–56^ racial/ethnic minorities,^25 27 28 31 42 57–59^ women living with HIV,^51 60 61^ sexual and gender minorities (lesbian and bisexual women, and transmales),^62 63^ adolescent girls and young women (AGYW),^54 63 64^ women of low socioeconomic status,^25 43 57 65^ and women experiencing homelessness.^29^

Nine studies employed a qualitative design, specifically in-depth interviews and focus group discussions, to explore women’s preferences related to HPV self-sampling.^24–32^ Of the nine qualitative studies, seven were conducted in high-income countries in Europe and North America^24–29 31^; some exclusively focused on vulnerable subpopulations such as ethnic minorities,^25 27 28 31^ rural women^26^ or women experiencing homeless.^29^ Six qualitative studies asked about hypothetical acceptability of HPV self-sampling. Two qualitative studies examined cervicovaginal self-sampling device preferences,^26 28^ two examined urine self-sampling^30 66^ and one examined self-sampling with a labial padette.^29^ Five studies employed a multimethod or mixed methods design.^47 67–70^ A total of 58 studies employed a quantitative approach: 47 cross-sectional studies,^41–46 48 50–63 65 66 71–94^ two pre–post surveys,^64 95^ eight RCTs^33–40^ and one prospective cohort.^49^ Quantitative studies examined a wide range of end users, including underscreened and vulnerable subpopulations such as women living HIV,^51 60 61^ transmales^62^ and female sex workers.^40^ Of these studies, the majority included women above age 30 years, but several focused exclusively on the values of preferences of AGYW.^54 63 64^ Most (n=67) quantitative studies focused on end users in high-income countries, while only 15 quantitative studies were conducted in low-income or lower middle-income countries.

#### Quality assessment of included studies

Common issues with study quality were sampling/selection bias, lack of validation of survey measures and lack of transferability of values and preferences results. Twenty-seven articles reported sampling or selection bias as a limitation of the study.^26 27 29 35–37 39 40 43 46 52 54 57 59 61–64 66 70 83 87 89 91 93 95 96^ Furthermore, one study acknowledged that participants self-selected to participate with the knowledge that self-collection would be a critical component of the study, so there may have been some selection bias towards acceptability of the self-test.^43^ One study assessed test–retest reliability and content validity of acceptability measures,^64^ but psychometric properties of acceptability were not conducted consistently across studies. Thirty-two articles reported that their results were not transferable to the study population or entire geographic population.^25 26 29 35–40 46 53 57 59 62 64–67 70 72 75 77–81 87 88 91 93–95^ Other study quality concerns identified in this review included: no consideration of study limitations, preference bias due to patient–physician interaction, recall bias, social desirability bias, low response rate and high loss to follow-up.

### Values and preferences related to logistical aspects of self-sampling

#### Overall acceptability

End users generally found HPV self-sampling acceptable and/or expressed willingness to undergo future HPV testing using self-sampling.^24 26 27 30 32 34 36–39 41 42 44 46 47 50 53 54 56 58 60–63 66–69 71 73 74 79–85 87–93 95^ Self-sampling was generally highly acceptable regardless of age, income or country of residence.

#### Self-sampling versus clinician sampling

Across most of the studies asking end users’ preference regarding self-sampling versus clinician sampling for HPV DNA, most end users chose self-sampling.^29 35 36 42 43 52 54 56 58 59 62 65 68 69 71 74 77–86 88 89 91–93 95^ The most frequently cited reasons for preferring self-sampling over clinician sampling were less pain or physical discomfort,^29 31 32 34–37 39 43 44 52 57 58 65 70 71 73 77–80 86 88–91 93–95^ ease of use, convenience, ability to perform the test in private^28 29 31–33 36 39 42 46 52 57 59 62 74 77 81 89 90 94^ and less embarrassment or anxiety.^27 28 31 33 35 37 39 40 46 52 59 65 69 70 72 73 77–80 89–91 94 95^

In nine studies, end users preferred clinician sampling over self-sampling.^25 28 30 53 57 64 73 76 87^ Even among end users that preferred self-sampling, many lacked confidence in accuracy of self-sampled specimens.^24 25 27–29 33 35–37 39–41 47 49 52–55 57 59 62–66 69 70 73 78–80 85 94^ End users expressed greater confidence in the clinician’s ability to collect the specimen properly. These concerns were expressed in both high-income and low-income settings.

#### Setting for self-sampling

In 21 studies, end users self-sampled from their homes.^26 33 35–38 43 45 46 49 58 59 69 72 74 81 82 84 85 92 93^ When asked to compare self-sampling at home versus in a clinic, most end users expressed a preference to collect the sample at home.^32 33 44 58 66 68 74 91^ In high-income countries, samples collected at home were usually sent for processing using the postal service, whereas in some low-income and middle-income countries, self-sampled specimens were collected by community health workers conducting home visits for processing from the homes of participants rather than mail. For example, in the Netherlands, one participant expressed a preference for self-sampling at home because, ‘It can be done whenever you want, at ease and in a familiar environment’.^81^ In two studies, end users reported a clear preference for clinic-based testing with a physician over self-sampling at home.^57 70^ Reasons for preferring clinic-based testing were lack of confidence in ability to self-sample^57^ and ability to receive treatment if needed.^70^

#### Instructions and assistance

Other studies suggested strategies to increase end user’s confidence in performing self-sampling correctly.^47 55 70^ In clinical settings, end users cited the important role of a trusted healthcare provider in fielding questions and providing reassurance that sampling was done correctly.^27 30 47 70^ In addition, clear, step-by-step instructions with illustrations in the appropriate language were suggested as methods to facilitate self-sampling and improve end users’ confidence.^62 68 79 81^

#### Self-sampling devices

Studies examined different HPV self-sampling devices. The cervical swab was by far the most common and well accepted. Other devices tested included the lavage,^74 81 85^ cervical brush,^42–44 48 52 53 55 60 69 70 72 79 82 84 86–88 92 94 95^ tampon^32 34 49–51^ and labial padette.^29^ Acceptability of the labial padette was assessed among a sample of women experiencing homelessness in the USA. Most women found the labial padette to be an acceptable form of self-sampling and was preferred over clinician sampling using a cervical brush though some women were concerned about reliability of the self-sampled specimens.^29^

Several studies assessed women’s preferences for different self-sampling devices and acceptability was generally high for all devices.^26 28 32–34 49–51 54 67 80 89^ Acceptability of self-sampling using a cervical swab and tampon was compared with clinician sampling using a cervical brush in a sample of rural Gambian women.^50^ Though all three methods were found to acceptable by more than 70% of participants, there was a clear preference for self-sampling using the cervical swab (97.1%) over self-sampling using tampon (84.4%) and clinician sampling using cervical brush (72.4%). When shown examples of different devices, a sample of HIV-positive South African women rated the highest preference for the cervical brush (51%), while fewer women preferred the tampon (31%) or lavage sampler (18%).^51^

#### Urine self-sampling

Two studies examined acceptability of an alternative method for cervicovaginal HPV DNA self-sampling, urine self-sampling.^30 66^ In a sample of Micronesian women, 95% of participants said they were comfortable with the urine test compared with only 82% for the Pap smear. Despite higher ratings for urine self-sampling, more women preferred a clinician to perform a Pap smear (44.2%) over self-sampling (38.8%) because they valued the knowledge and expertise of the clinician.^30^ In a study of Swedish women with CIN, 85% of participants rated self-sampling procedures (urine and cervical swab) as easy, and 74% said they could see themselves self-sampling at home.^66^ The study did not compare women’s preferences for the swab versus urine self-sampling.

### Values and preferences by end user characteristics

#### Adolescent girls and young women

Three studies focused solely on the values and preferences of AGYW.^54 63 64^ In an online survey of lesbian and bisexual AGYW in the USA, 51% participants reported willingness to self-sample at home. Willingness increased with age and concern about getting an HPV-related disease.^63^ The other two AGYW-focused studies assessed HPV self-sampling acceptability and preferences after participants performed self-sampling.^54 64^ Among South African students aged 16–22 years, 56% preferred self-sampling (56%) over clinician sampling.^54^ Among AGYW attending a teen health centre in the USA, acceptability for both self-sampling and clinician sampling was high. However, most participants expressed a preference for clinician sampling.^63^

#### Generational differences among end users

Twenty-five studies compared differences in sampling preferences by age group.^24 31 33–35 39–41 49 52 55 59 65 67 69 72–74 76 77 79 82 89 91 95^ Of these, 14 studies found no differences in preferences for self-sampling versus clinician sampling comparing older versus younger women.^35 39 41 52 55 59 65 69 73 76 79 82 89 91^ Studies presenting preferences or attitudes adjusted for sociodemographic and/or behavioural characteristics were more likely to find no difference in preference for self-sampling by age group.^69 76 89^ Five studies reported greater preference for self-sampling among younger age groups compared with older age groups.^31 33 34 72 95^ In one study, Dutch young women cited reasons of decreased embarrassment, time/effort investment and a ‘do-it-yourself’ attitude more often than older women when describing why they preferred HPV self-sampling over clinician sampling.^33^ Other studies found that older women were more likely to prefer self-sampling over clinician sampling.^24 40 42 63^ A qualitative study of women in Switzerland found that younger women were used to visiting a gynaecologist and did not see the necessity of changing this practice, while some older women, less used to regular gynaecological appointments, were more in favour of self-sampling, especially if they previously had had a negative experience with pelvic examinations.^24^

#### Geographic region

Only one study compared end users’ preferences across different regions and countries.^70^ The study found that women in rural Uttar Pradesh (93.1%), Hyderabad (95.5%), Uganda (64.5%) and Nicaragua (50.0%) preferred self-sampling over clinician sampling. More than half of surveyed respondents in each site reported self-sampling as ‘easy’ with the lowest percentage in Hyderabad (53.6%) and the highest percentage in Uganda (89.7%). Women across all study sites preferred to conduct self-sampling in a clinic setting rather than at home so that a provider could clarify questions or do the test if needed.

#### Women living with HIV

Three studies examined HPV sampling preferences among end users living with HIV.^51 60 61^ One study surveyed a sample of HIV-positive and HIV-negative women from northern Brazil, reporting that 87% of women found cervicovaginal sampling for HPV DNA acceptable. A greater proportion of HIV-positive women reported acceptability (97%) than HIV-negative women (84%).^60^ A study of HIV-positive urban and rural women in South Africa compared preferences for cervical brush, tampon and lavage sampling devices.^51^ HIV-positive rural women preferred the cervical brush over other sampling devices, whereas urban women preferred the tampon over other devices.

#### Sexual and gender minorities

Two studies conducted in the USA examined preferences among sexual and gender minorities. As mentioned previously, more than half of lesbian and bisexual AGYW surveyed reported willingness to self-sample at home.^63^ Among transmales who had undergone both self-sampling and clinician sampling for HPV DNA, 90% expressed a preference for self-sampling citing privacy, ease and self-empowerment as reasons for their preference. Some participants cited feelings of gender dysphoria and difficulty positioning the body or swab for self-sampling as challenges of self-sampling.^62^

## Discussion

In this systematic review, we found general consensus that self-sampling is a highly acceptable method for HPV testing, regardless of study location and sampling method, device, setting or participant demographics. Our findings align with previous reviews, such as one by Nelson *et al*, which reported a pooled estimate prevalence of women preferring self-sampling of 59% (95% CI 48% to 69%).^13^ Similarly, high levels of acceptability were consistent among vulnerable and underscreened subpopulations. Women generally preferred self-sampling over clinician sampling, citing ease of use, privacy, convenience and physical and emotional comfort as major reasons for their preference. Women who preferred clinician sampling expressed concerns about the reliability of self-sampled specimens, which was also the most common reason women reported for disliking self-sampling in the review by Nelson *et al*.^13^ Another common reason identified uniquely in this review is end user concern that they would not get face time with a clinician if needed. Counselling prior to the invitation to self-sample as well as clear instructions and availability of trustworthy clinical staff to assist with self-sampling could remedy some of these concerns.

A number of studies examined acceptability of self-sampling in different settings, such as the clinic, home or community. More than half of home-based self-sampling studies took place in high-income countries, since a quick and reliable postal system was necessary for mailing the samples for processing. In some low-income and middle-income countries, community health workers collected specimens self-sampled by participants for processing during home-visits. Across studies, the home was regarded as a highly acceptable and convenient setting for self-sampling. However, women expressed that they would prefer clinic-based sampling if home-based sampling meant they would not have access to a healthcare provider. Ensuring that women have access to a healthcare provider to answer questions about screening or a potential positive result is an important consideration for the expansion of home-based testing programmes.

The most common device for self-sampling was the cervical swab. Among studies comparing acceptability of different self-sampling devices, end users preferred the cervical swab over other devices such as the lavage or cervical brush, but this preference could be due to greater familiarity with cervical swab. Studies examining less commonly used devices (eg, tampon and labial padette) and methods (eg, urine sampling) generally found high acceptability; however, only 15 studies examined these alternative self-sampling devices and methods. Therefore, preference for self-sampling device and method is an important area for further study.

This review identifies a need for further research examining women’s preferences in low-income countries, which bear a disproportionate burden of new cervical cancer cases. Only 3 of the 72 studies included in this review were conducted in low-income countries, leaving a critical gap in understanding of end users’ preferences in these settings. HPV DNA self-sampling is a promising screening method that, when offered to women in low-resource settings, can address barriers that create inequalities in access to cervical cancer screening.^97^

### Strengths and limitations

This is one of the first studies to systematically review both qualitative and quantitative literature on values and preferences for HPV self-sampling for cervical cancer screening. We synthesised end users’ values and preferences related to the setting of sample collection and the device used to collect the sample. We also examined values and preferences for HPV self-sampling among vulnerable populations.

Findings from this review should be viewed in light of its limitations. We did not include conference abstracts or grey literature in this review, so our findings may not fully represent of the full body of literature on values and preferences for HPV self-sampling. Given the diversity of measures used to assess values and preferences, it is difficult to make comparisons across studies and therefore difficult to determine the specific aspects of self-sampling that clients find acceptable and whether these hold true for clients in different age groups, geographic regions and socioeconomic status.

## Conclusion

WHO strongly supports inclusion of self-sampling for HPV testing as an additional approach to sampling in cervical cancer screening programmes where HPV tests are used. HPV self-sampling is generally a highly accepted method of cervical cancer screening for women across the world. This systematic review of values and preferences builds on previous reviews on self-sampling acceptability. Understanding women’s preferences for HPV self-sampling is a critical factor for expanding choice, coverage and uptake of cervical cancer screening. This will be critical to reach WHO’s target of 70% cervical cancer screening coverage by 2030.^6^ Screening with high uptake will expedite reductions and will be necessary to eliminate cervical cancer in countries with the highest burden.^98^

## Data Availability

Data are available on request. All data relevant to the study are included in the article or uploaded as supplementary information. Extracted data are available on request to the corresponding author.
